# Hydrothermal Fabrication of WO_3_ Hierarchical Architectures: Structure, Growth and Response

**DOI:** 10.3390/nano5031250

**Published:** 2015-07-22

**Authors:** Chuan-Sheng Wu

**Affiliations:** 1 State Key Laboratory of Coal Mine Disaster Dynamics and Control, Chongqing University, Chongqing 400044, China; 2 Mining Engineering Post-Doctorate Mobility Station of Chongqing University, Chongqing 400044, China; 3 International College of Chongqing Jiaotong University, Chongqing 400074, China; E-Mail: cqucswu@aliyun.com; Tel.: +86-23-65336832; Fax: +86-23-65336832

**Keywords:** WO_3_, crystal growth, functional, CO, sensors

## Abstract

Recently hierarchical architectures, consisting of two-dimensional (2D) nanostructures, are of great interest for potential applications in energy and environmental. Here, novel rose-like WO_3_ hierarchical architectures were successfully synthesized via a facile hydrothermal method. The as-prepared WO_3_ hierarchical architectures were in fact assembled by numerous nanosheets with an average thickness of ~30 nm. We found that the oxalic acid played a significant role in governing morphologies of WO_3_ during hydrothermal process. Based on comparative studies, a possible formation mechanism was also proposed in detail. Furthermore, gas-sensing measurement showed that the well-defined 3D WO_3_ hierarchical architectures exhibited the excellent gas sensing properties towards CO.

## 1. Instructions

It is well known that the morphological characteristics of the materials such as grain size and structure play a crucial role in affecting their chemical and physical properties, and the morphology strongly depends on the preparation method and condition. Recently, the synthesis of complex three-dimensional (3D) micro/nanoarchitectures with distinct structural and geometrical features such as arrays [1], networks [2], and hierarchical structures [3] has gathered immense interest because of their inherent anisotropic nature and tunable spatial distribution. This way may provide a great deal of opportunity to explore their novel properties and application. At the meantime, integration of nanorod/nanowire/nanosheet as building blocks into hierarchical architectures were successfully performed [4,5,6]. These hierarchical architectures are useful for the realization of functional nano-devices because the aspect ratio and surface to volume ratio of these materials are extremely high [7,8].

As an important wide gap semiconductors, tungsten oxides (WO_3_) have many outstanding properties resulted in varied applications in many areas, especially gas sensors [9] and catalysis [10,11]. Nowadays, a great deal of efforts have been focused on the exploration of new routes for the preparation of 1D/2D WO_3_ nanostructures [9,12,13,14,15,16]. However, fabrication of more complex nanoarchitectures and controlling the shape of nanostructures at the microscopic level still remains a significantly challenging task.

In this present work, we report a novel synthesis of 3D rose-like WO_3_ hierarchical architectures under relative mild conditions. The as-prepared WO_3_ hierarchical architectures were in fact assembled by numerous nanosheets. Based on comparative studies, the growth mechanism was elaborated in detail. Furthermore, the gas-sensing performances of the as-prepared WO_3_ hierarchical architectures were investigated towards CO.

## 2. Experimental

In a typical procedure, 0.1 mol Sodium tungstate (Na_2_WO_4_·2H_2_O) and 0.25 mol oxalic acid (H_2_C_2_O_4_) were mixed into 100 mL of deionized water under an electromagnetic stirring. Hydrochlolic acid (HCl, 4 M) was dropped into the above transparent solution under continuous stirring until the pH was 2, and then the solution was transferred into a 100 mL autoclave, which was put into an electrothermic oven and heated to 150 °C from room temperature. After 6 h of reaction, yellow precipitates were obtained. Then the as-prepared precipitates were washed several times with deionized water and ethanol. After 4 h of dried and annealed at 400 °C in air, rose-like WO_3_ hierarchical architectures were obtained.

The as-prepared product was characterized by the X-Ray Diffraction (XRD, Rigaku D/Max-1200X, Tokyo, Japan), the Scanning Electronic Microscopy (SEM, Nova 400 Nano, Salem, OR, USA) and transmission electron microscope (TEM, ZEISS, LIBRA200, Braunschweig, Germany) with an accelerating voltage of 200 kV.

The gas sensors were fabricated using the obtained WO_3_ powders. In detail, the powders were further dispersed in the ethanol and ultrasonicated into slurry suspension, and then it was coated onto the surface of an Al_2_O_3_ ceramic tube by a small brush to form a thickness of 10~20 μm film between two parallel Au electrodes, which had been previously printed at the both end sides of the tube. The distance between two electrodes was estimated to be 6 mm and the diameter of the tube was 1.2 mm. A resistor wire coil was inserted in the tube as a heater, the role of which was controlling the working temperature of the sensor.

Gas sensing properties were measured using a static system (HW-30A, Hanwei Electronics Co., Zhengzhou, China) that controlled by a computer. Sensor devices were loaded inside a small custom-built gas flow chamber with low dead volume. Before gas sensing testing, the fresh sensors should be aged for 3 days. As a typical reducing gas, the response in this paper was defined as *S* = *R_g_*/*R_a_*, where which *R_a_* and *R_g_* are the resistance of the sensor in air and in CO gas, respectively. And the response and recovery time was counted as the time taken to reach 90% of the total resistance change in the case of adsorption and desorption, respectively.

## 3. Results and Discussion

**Figure 1:**
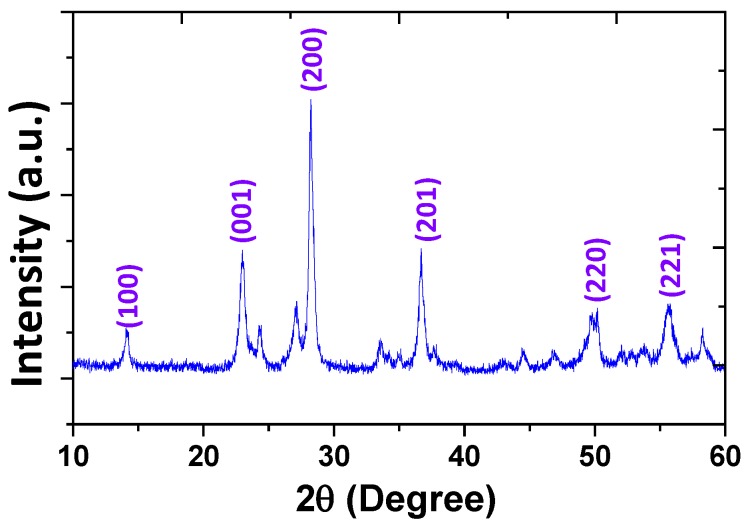
XRD spectra of the as-prepared rose-like WO_3_ hierarchical architectures.

**Figure 2:**
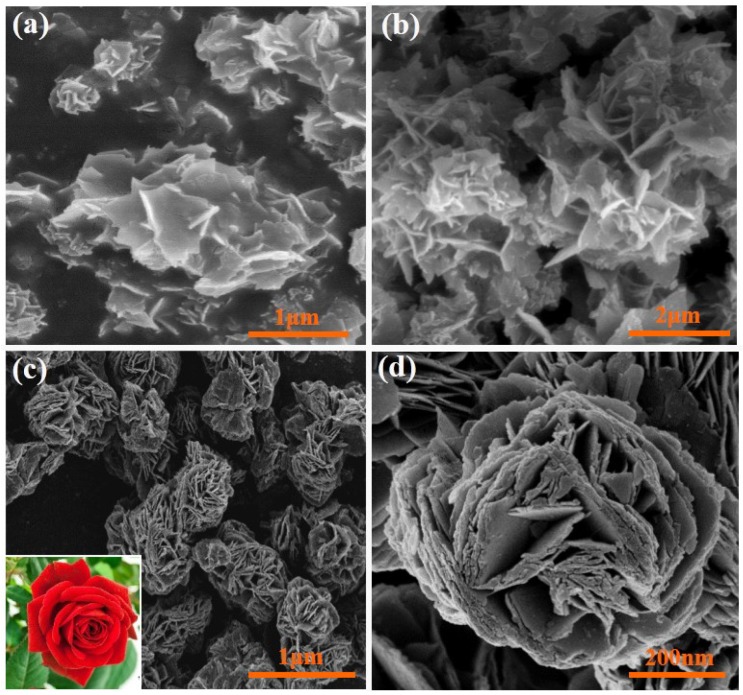
SEM images of the samples prepared with various molar mass of H_2_C_2_O_4_: (**a**) 0 mol, (**b**) 0.1 mol, (**c**) 0.25 mol and (**d**) the corresponding magnified image of (**c**).

Figure 1 shows a typical XRD pattern for the as-prepared rose-like WO_3_hierarchical architectures. The results show that the peaks are sharp and strong, signifying the high-degree crystallization of the samples. All diffraction peaks of the samples can be indexed to a pure hexagonal WO_3_ (JCPDS Card No. 33-1387). No remarkable shift in diffraction peak detected, which indicates that no intermediate products are produced during reaction. The SEM images of the three different samples are shown in Figure 2. When there is no oxalic acid during preparing, as we can see from Figure 2a, a small amount of irregular nanosheets with various sizes are obtained. The surface of these nanosheets are smooth and thickness of them is about 20–30 nm. However, the morphologies of the samples change dramatically after addition of H_2_C_2_O_4_ as structure-directing agent. When the 0.1 mol of H_2_C_2_O_4_ was added, many nanosheets appear and these nanosheets begin to fit together (Figure 2b). However, when the molar mass of H_2_C_2_O_4_ increased to 0.25, as presented in Figure 2c, numerous nanosheets are assembled to form 3D rose-like architectures with uniform diameters ~500 nm. No other morphologies emerge, indicating a high yield of these rose-like architectures. The above observations indicate that the presence of H_2_C_2_O_4_ plays a role in gathering these nanosheets to form 3D assembled rose-like architectures in current experiment. By adjusting the amount of oxalic acid, the control of precipitation between WO4^2−^ and H^+^ is achieved, which ensures the formation of small and uniform crystal seeds in strongly acidic solution. Without or few H_2_C_2_O_4_, only irregularly aggregated textures have been generated.

**Figure 3:**
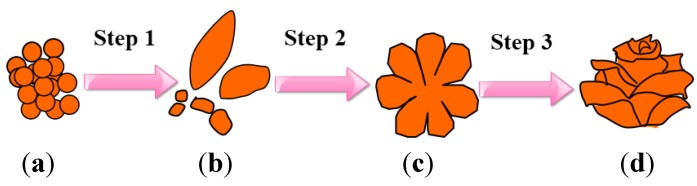
Schematic illustration of the possible formation processes of rose-like WO_3_; hierarchical architectures.

The possible formation process of 3D WO_3_ hierarchical nanostructures is illustrated in Figure 3. At the initial stage of hydrothermal treatment, numerous nanoparticles are produced because high concentration is suitable for the rapid formation of small crystallites due to the domination of kinetic factor. (Step 1) When the hydrothermal time prolonged, the primary small crystallites will occur self-aggregate during the reaction and then grow into large nanoparticles or nanosheets through dissolution and recrystallization (as shown in Figure 3b) [17]. With the further increase of the hydrothermal time, the stacking and organizing of nanosheets by dipole-dipole interactions and oriented attachment coupled simultaneously with their further growth results in the formation of primary flower-like assemblies (Step 2, as shown in Figure 3c) [18]. Finally, the progressive self-assembly and -modification process occurs in the synthetic solution resulting in the ultimate rose-like hierarchical architectures (Step 3, as shown in Figure 3d).

**Figure 4:**
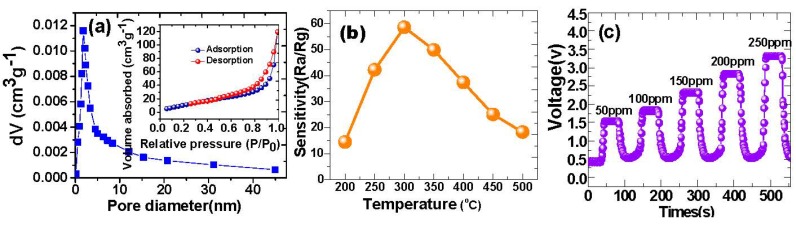
(**a**) Pore-size distribution curve and nitrogen adsorption and desorption isotherms (inset) of the rose-like WO_3_ hierarchical architectures; (**b**) Sensitivity of the sensor to the CO at temperatures from 200 to 500 °C; and (**c**) Response of the sensor to different concentrations of CO.

Figure 4a shows the N_2_ adsorption-desorption isotherm of the WO_3_ hierarchical nanostructures. The BET (Brunauer Emmett Teller) specific surface area is 78.2 m^2^/g, the value of which is much larger than that of the hierarchically porous WO_3_ microflower (13.1 m^2^/g) [17] and WO_3_ powder (58.5 m^2^/g) [19]. In addition, the pore size distribution diagram (the inset of Figure 4a) based on Barretl-Joyner-Halenda (BJH) method show that the average pore diameter is 13.8 nm. These results indicate that the rose-like WO_3_ hierarchical structures have a large specific surface area which is mainly due to the existence of numerous mesoporous. Figure 4b shows gas-sensing response of the WO_3_ hierarchical structures to CO (200 ppm) as a function of the working temperature ranging from 200 to 500 °C. The optimal gas response is estimated to be 58.6 at the operating temperature of 300 °C. And as shown in Figure 4c, the response amplitude of the WO_3_ hierarchical nanostructures is significantly increased with increasing CO concentration. Moreover, a comparison with WO_3_ powder (*S* = 2.5 at 100 °C, 500 ppm CO) [20] and WO_3_ film (*S* = 6.67 at 200 °C, 200 ppm CO) [19] indicate that the rose-like WO_3_ hierarchical structure we prepared exhibit superior gas-sensing properties (*S* = 15 at 200 °C, 200 ppm CO), and represent an advance of hierarchical nanostructures in further enhancing the functionality of gas sensors.

## 4. Conclusions

Hexagonal rose-like WO_3_ hierarchical architectures with largest amount of petals and pores were synthesized through a facile hydrothermal method in the presence of oxalic acid. The hierarchical architectures were interwoven with numerous single crystal nanosheets, forming a rose-like porous structure, and resulted in a high specific surface area. The oxalic acid plays a critical role in determining the ultimate morphologies of WO_3_ nanostructures. The gas-sensing measurements reveal that the sensor made of the WO_3_ hierarchical architectures shows excellent gas response to CO gas.

## References

[1] Song L.M., Zhang S.J., Wu X., Wang Z., Wei Q.W. (2013). One-step synthesis, growth mechanism, and optical properties of 3D YIO_3_ hollow microspheres consisting of nanotube arrays. Powder Technol..

[2] Chen J.K., Gui X.C., Wang Z.W., Li Z., Xiang R., Wang K.L., Wu D.H., Xia X.G., Zhou Y.F., Wang Q. (2011). Superlow thermal conductivity 3D carbon nanotube network for thermoelectric applications. ACS Appl. Mater. Interface.

[3] Wang W.S., Zhen L., Xu C.Y., Yang L., Shao W.Z. (2008). Room temperature synthesis of hierarchical SrCO_3_ architectures by a surfactant-free aqueous solution route. Cryst. Growth Des..

[4] Chang C.M., Hon M.H., Leu I.C. (2012). Improvement in CO sensing characteristics by decorating ZnO nanorod arrays with Pd nanoparticles and the related mechanisms. RSC Adv..

[5] Chen D., Xu J., Xie Z., Shen G.Z. (2011). Nanowires assembled SnO_2_ nanopolyhedrons with enhanced gas sensing properties. ACS Appl. Mater. Interface.

[6] Li J., Fan H.Q., Jia X.H. (2010). Multilayered ZnO nanosheets with 3D porous architectures: Synthesis and gas sensing application. J. Phys. Chem. C.

[7] Hu H., Yu L., Gao X.H., Lin Z., Lou X.W. (2015). Hierarchical tubular structures constructed from ultrathin TiO_2_ nanosheets for highly reversible lithium storage. Energy Environ. Sci..

[8] Yu X.Y., Hu H., Wang Y.W., Chen H.Y., Lou X.W. (2015). Ultrathin MoS_2_ nanosheets supported on N-doped carbon nanoboxes with enhanced lithium storage and electrocatalytic properties. Angew. Chem. Int. Edition.

[9] Hieu N.V., Quang V.V., Hoa N.D., Kim D. (2011). Preparing large-scale WO_3_ nanowire-like structure for high sensitivity NH_3_ gas sensor through a simple route. Curr. Appl. Phys..

[10] Li Y., Hsu P.C., Chen S.M. (2012). Multi-functionalized biosensor at WO_3_–TiO_2_ modified electrode for photoelectrocatalysis of norepinephrine and riboflavin. Sens. Actuators B Chem..

[11] Krüger P., Koutiri I., Bourgeois S. (2012). First-principles study of hexagonal tungsten trioxide: Nature of lattice distortions and effect of potassium doping. Phys. Rev. B.

[12] Sonia A., Djaoued Y., Subramanian B., Jacques R., Eric M., Ralf B., Achour B. (2012). Synthesis and characterization of novel nanorod superstructures and twin octahedral morphologies of WO_3_ by hydrothermal treatment. Mater. Chem. Phys..

[13] Park S., Kim H., Jin C., Choi S., Kim S.S., Lee C. (2012). Enhanced CO gas sensing properties of Pt-functionalized WO_3_ nanorods. Thermochim. Acta.

[14] Xiang Q., Meng G.F., Zhao H.B., Zhang Y., Li H., Ma W.J., Xu J.Q. (2010). Au nanoparticle modified WO_3_ nanorods with their enhanced properties for photocatalysis and gas sensing. J. Phys. Chem. C.

[15] Sun S.B., Chang X.T., Li Z.J. (2012). Growth study and photocatalytic properties of Co-doped tungsten oxide mesocrystals. Mater. Charact..

[16] Lou X.W., Zeng H.C. (2003). An inorganic route for controlled synthesis of W_18_O_49_ nanorods and nanofibers in solution. Inorg. Chem..

[17] Huang J., Xu X.J., Gu C.P., Fu G., Wang W., Liu J. (2012). Flower-like and hollow sphere-like WO_3_ porous nanostructures: Selective synthesis and their photocatalysis property. Mater. Res. Bull..

[18] Yu J., Qi L.F. (2009). Template-free fabrication of hierarchically flower-like tungsten trioxide assemblies with enhanced visible-light-driven photocatalytic activity. J. Hazard. Mater..

[19] Susanti D., Kusuma G.E., Muttaqin A. (2012). Synthesis and application of WO_3_ as material for poisonous CO gas sensor. Prosiding InSINas.

[20] Susanti D., Perdana A.S., Purwaningsih H., Noerochim L., Kusuma G.E. (2014). Preparation of CO gas sensor from WO_3_ nanomaterial synthesized via sol-gel method followed by hydrothermal process. AIP Conf. Proc..

